# Herbivores Coprolites from Chehrabad Salt Mine of Zanjan, Iran (Sassanid Era, 224–651 AD) Reveals Eggs of Strongylidae and Anoplocephalidae Helminths

**Published:** 2020

**Authors:** Masoumeh MEIGOUNI, Mahsasadat MAKKI, Ali HANILOO, Zeynab ASKARI, Iraj MOBEDI, Saied Reza NADDAF, Nicole BOENKE, Thomas STOLLNER, Abolfazl AALI, Zahra HEIDARI, Gholamreza MOWLAVI

**Affiliations:** 1. Department of Parasitology and Mycology, School of Medicine, Zanjan University of Medical Sciences, Zanjan, Iran; 2. Department of Medical Parasitology and Mycology, School of Public Health, Tehran University of Medical Sciences, Tehran, Iran; 3. Department of Parasitology, Pasteur Institute of Iran, Tehran, Iran; 4. Ruhr Universität Bochum, Institut für Archäologische Wissenschaften, Am Bergbaumuseum 31, D-44791 Bochum, Germany; 5. Archaeological Museum of Zanjan, Zanjan, Iran; 6. Department of Medical Microbiology and Parasitology, School of Medicine, Ardabil University of Medical Sciences, Ardabil, Iran; 7. Center for Research of Endemic Parasites of Iran (CREPI), Tehran University of Medical Sciences, Tehran, Iran

**Keywords:** Paleoparasitology, Herbivores, Strongyle, Anoplocephalan, Iran

## Abstract

**Background:**

The ancient Chehrabad Salt mine, a well-known archaeological site in Iran, has recently received increasing interest from Iranian and international archeologists. Also, the biological remains from this site have provided valuable sources for studying the pathogenic agents of ancient times. This study aimed to identify the parasitic helminth eggs preserved in the herbivores coprolites.

**Methods:**

From 2011 to 2015, we received three coprolites belonging to herbivorous animals recovered during excavations in Chehrabad Salt mine of Zanjan, Iran. The coprolites were dated back to the Sassanid era (224–651 AD) by using radiocarbon accelerator mass spectrometry (AMS) and archeological stratigraphy methods. Following rehydration of the specimens in a 0.5% trisodium phosphate solution, the suspensions were mounted in glycerin jelly on glass slides and examined by a light microscope with 100x and 400x magnifications.

**Results:**

Two coprolites belonged to donkeys and one to an unknown herbivore species. The recovered eggs belonged to members of two helminths families, Strongylidae, and Anoplocephalidae. Also, within the two coprolites, some mites, presumably of the order Oribatida, were observed.

**Conclusion:**

The presence of two different nematodes in the equids coprolites provide clues of the burden of helminths infection on working animal at the Sassanid time and demonstrates the appropriate preservation condition of biological remains in the ancient salt mine of Chehrabad as well.

## Introduction

The parasites in the ancient biological remains provide an image of the status of parasitic infections in a specific period in the past. The coprolites are a valuable source for tracing parasitic agents in humans and animals over time. They can as well shed light on the emergence and elimination of parasitic infections in time and provide clues on the intercontinental migrations of humans and animals (1).

The parasitic worms of equids such as strongyles have a worldwide distribution (2–6), while the available data of their presence in ancient times is very scarce (7, 8). Strongyle nematodes are amongst the most prevalent pathogenic helminth inhabiting the large intestine of herbivores. These nematodes are soil-transmitted helminths and are acquired by the animals via the ingestion of the third-stage larvae (L3). Infection with this parasite produces mild to severe clinical symptoms due to the migration of the larvae (9, 10). In contrast to strongyles, the Anoplocephalidae cestodes are arthropod-borne helminths and infect the hosts via the ingestion of oribatid mites (11). Previously, in Chehrabad salt mine archeological site, eggs of several helminths that infect humans, rodents, and carnivores were identified (12–14). The appropriate preservation condition in this ancient site has preserved biological remains over the past millennia. The present study describes the identification of the helminth eggs in herbivores coprolites recovered from Chehrabad Salt mine archaeological site.

## Materials and Methods

### Samples

From 2011 to 2015, we received three coprolites (Code numbers: 2605–286, 2357–282, and 2462–124) from the Cultural Heritage Organization of Zanjan, northwestern Iran. Of three coprolites, two (code numbers 2605–286 and 2462–124) matched donkey droppings based on the size and their typical round (cubic) kidney shape. The third sample that had lost its original shape was attributed to a herbivore by archaeobotanical analysis.

The coprolites were dated back to the Sassanid era (224–651AD) by using radiocarbon accelerator mass spectrometry (AMS) and archeological stratigraphy methods as described elsewhere (15,16). The samples were kept in the Laboratory of Helminthology at the School of Public Health, Tehran University of Medical Sciences, until used.

### Microscopical examination

The samples were rehydrated in a 0.5% trisodium phosphate solution (Na3Po4) for one week (17, 18). The suspension from each specimen was mounted in glycerin jelly on 200 glass slides and examined under a light microscope at 100X and 400X magnifications. The retrieved eggs were photographed by a camera-equipped microscope (Labomed LX 500, Springfield, New Jersey, USA); their measurements were recorded and compared with similar eggs available in the literature. The helminths’ eggs were identified based on the measurements and morphological characters available in taxonomic keys (19, 20).

## Results

**Fig. 1:**
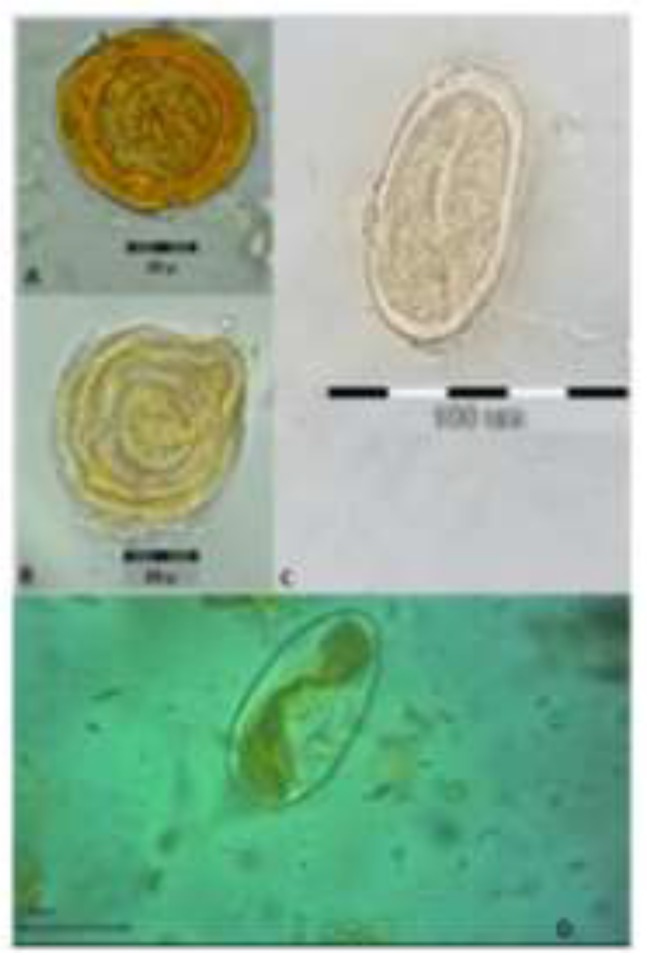
Recovered helminth eggs from donkeys (A, B&C) and unidentified herbivore (D) coprolites. Strongylidae eggs (C&D) with larvae inside, and Anoplocephalidae eggs (A&B).

Based on reliable morphological features and measurements, we identified two types of eggs belonging to the members of the families Strongylidae and Anoplocephalidae (Fig. 1). The typical thick shells with the pyriform apparatus inside, demonstrated Anoplocephalidae eggs (Fig 1. A&B), and the thin oval shell, containing larvae, clearly represented the strongyles’ eggs (Fig. 1, C&D) (19, 20).

**Fig. 2:**
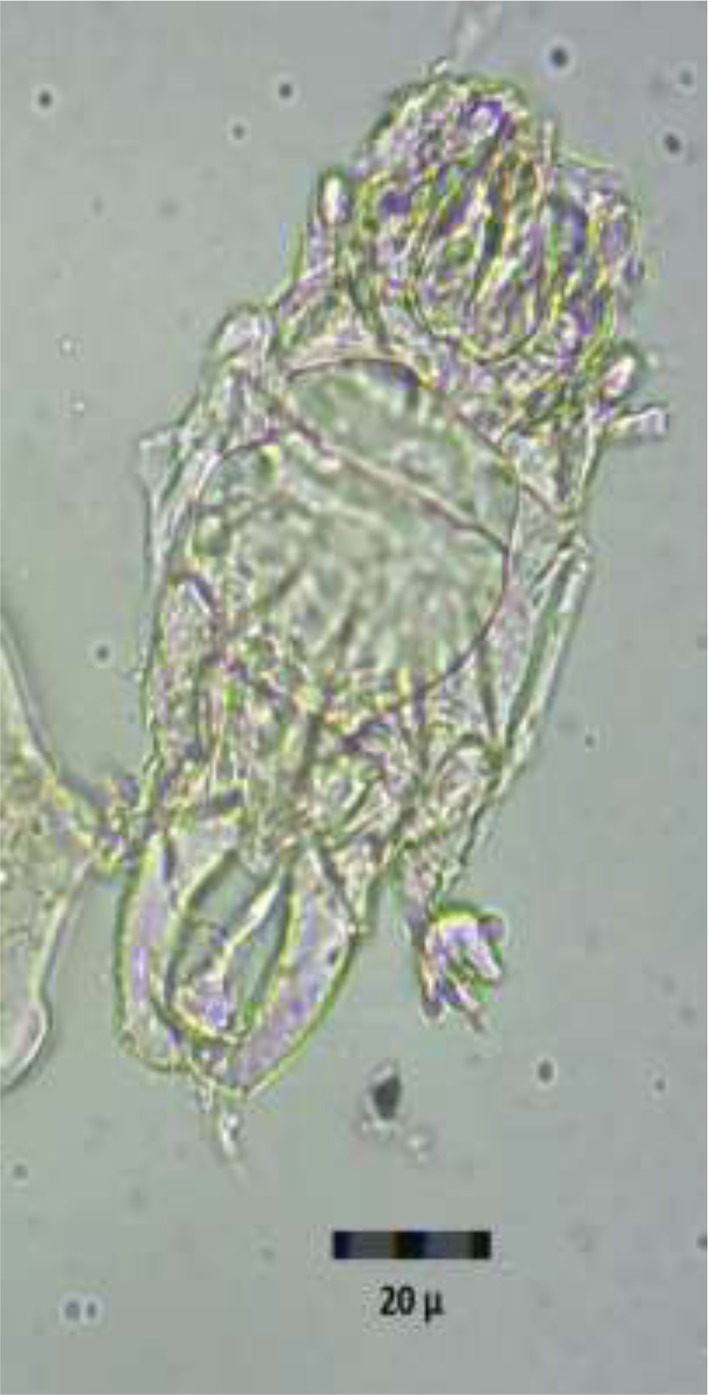
The mite in herbivores coprolites recovered in Chehrabad Archeological Site, Zanjan, Iran.

The specimen with the code number 2605–286 contained both types of helminths eggs, while the specimens with the code numbers 2357–282 and 2462–124 harbored only the eggs of Strongylidae and Anoplocephalidae, respectively (Table 1). Also, within the two coprolites with code numbers 2605–286 and 2462–124, mites of the class Arachnida, subclass Acari were observed. (Fig. 2).

**Table 1:** Helminth eggs retrieved from the herbivores coprolites

***Code number***	***Anoplocephalidae eggs***	***Strongylidae eggs***
2605–286	**+**	**+**
2357–282	−	**+**
2462–124	**+**	−
Egg measurements (μm)	53.6±3.2	72.30±7.09
(length and width range mean)	53.4±1.9	37.35±5.53

## Discussion

The coprolites recovered from archaeological sites are valuable sources for identification of the parasites that were prevalent in ancient times; however, well-preserved coprolites are not much available (7, 8). The present study reports the identification of two helminths eggs in the well-preserved ungulate coprolites from Chehrabad salt mine of Zanjan, North West of Iran. The eggs belonged to the members of families, Strongylidae and Anoplocephalidae, today commonly found in the digestive tracts of the equines.

The presence of mites within the coprolites drags the attention to oribatid mites that might serve as intermediate hosts of *Anoplocephala* tapeworms of cattle or other ruminants. These mites belong to the superorder Acariformes and commonly occur in soil and humus. Today, equine strongylosis is highly prevalent worldwide (3, 21, and 22) and poses a serious threat to the populations of horses and donkeys (4, 10). In Iran, in Marand, an area adjacent to the Chehrabad Archeological Site, the prevalence rate of strongyle nematodes among 58 donkeys was 100% (23, 24). The occurrence of strongyles among the equids does not pose a public health threat; however, very few reports on human infections with *Bertiella studeri,* a member of the Anoplocephalidae family, are available (25–27).

The infection with some strongyle species, e.g., *S. vulgaris*, may result in the mesenteric arterial obstruction in donkeys (5, 28). Hence, equine strongylosis in the Sassanid era might have been a significant veterinary problem or even a fatal disease of working animals; the only means of transportation in ancient times (29). Individual animals commonly harbor multiple species, which may apply to the coprolites we examined; precise identification of the species by morphological features of eggs and early-stage larvae is impossible. The inter-specific differences in rDNA sequences of *Strongylus* spp. allows the development of reliable molecular tools to differentiate individual eggs (30–33). In addition, the capability of the real-time PCR assay, which detects equivalents of 0.5 strongyle eggs with no cross-reactivity (34), increases the chance of finding helminth eggs in a limited amount of coprolites.

Molecular characterization of the ancient DNA of helminths provides a comparison of the archaic and modern populations of the helminth parasites and elucidates the evolution of parasites over millennia and the coevolution of host and parasite (35, 36).

## Conclusion

The presence of two different helminth eggs in the coprolites of equids provide clues of the burden of helminths infection on working animal at the Sassanid time and demonstrates the appropriate preservation condition of biological remains in the ancient salt mine of Chehrabad.
